# The cognitive processing of numerical bases: a review of empirical evidence

**DOI:** 10.1098/rstb.2024.0221

**Published:** 2025-10-20

**Authors:** Silke M. Göbel, Matthew Inglis, Julia Bahnmueller

**Affiliations:** ^1^ Department of Psychology, University of York, York YO10 5DD, UK; ^2^ Centre for Research on Equality in Education (CREATE), University of Oslo, 0371 Oslo, Norway; ^3^ Centre for Mathematical Cognition, Loughborough University, Loughborough LE11 3TU, UK

**Keywords:** transparency of base, number word inversion, binary, hexadecimal, artificial number symbols, decimal

## Abstract

We review the empirical research on numerical bases in the context of spoken and written numerical expressions, visual number symbols and the mappings between these expressions and symbols. Most of this research has been carried out with speakers of Indo-European languages, which have decimal systems. Children typically acquire spoken numerical expressions before they learn associated visual number symbols, but numerical expressions are language-specific. The structure of the numerical expressions influences number word learning, with evidence that an explicit base-10 structure in the spoken numerical expressions (i.e. *base transparency*) is advantageous. Later, when visual base-10 number symbols are acquired, inconsistencies in the mapping between those and the numerical expressions affect numerical and mathematical learning. We also review research on base-20 numeration systems and on inversion in number words: when the order of constituents in the spoken number word is inversed in relation to their order in the visual number symbols, e.g. ‘fourteen’ versus ‘14’. In conclusion, we discuss evidence about how using number symbols with a non-decimal base affects the processing of numbers and make suggestions for future research.

This article is part of the theme issue ‘A solid base for scaling up: the structure of numeration systems’.

## 1. Introduction

Most human adults in the Western world use symbols for exact numbers (e.g. Western numerals such as ‘25’ and spoken or written number words, from now on called ‘numerical expressions’, such as ‘twenty-five’) with ease in everyday life. But both formats, written numerals and numerical expressions, are cultural inventions that must be learnt. Our goal in this article is to review work from the mathematical cognition literature relevant to how this learning is influenced by numerical bases.

The main focus will be on the lack of transparency of key elements, especially with respect to numerical base, and the incongruency across different formats, especially with respect to the order of numerical constituents. We will focus on two formats, numerals and numerical expressions. One key question in the field is whether an explicit representation of a numerical base in numerical expressions helps in learning numerical concepts. Most of this research has been carried out with speakers of Indo-European languages who typically use a decimal verbal system and Western numerals.

After briefly introducing terminology and three influential theoretical accounts of number processing, our review consists of four main sections. We first describe research which shows that learning of numerical expressions is influenced by whether the base is named explicitly in a numerical expression (*base transparency*). Next, we discuss research which shows that the extent to which individuals can fluently map between numerals and numerical expressions is influenced by both base transparency and the congruency of order, that is, whether building blocks in numerical expressions (e.g. ‘vierundzwanzig’) and numerals (‘24’) are ordered in the same way. We then review the educational implications of this body of work. Finally, we review the limited empirical work which has explored the cognitive issues involved in learning, and operating with, non-decimal bases. Throughout, we emphasize how individuals’ numerical cognition is influenced by their prior knowledge of, and experience with, numbers, and that this can be seen as the underpinning cause of many of the issues we discuss. Before concluding, we discuss avenues for further research, and emphasize that theoretical accounts of numerical cognition should move beyond the implicit assumption that learners are monolingual and are familiar with just one system, i.e. that they are monosystem.

*Numerals*. We use the term ‘numerals’ here to refer to visually accessed non-linguistic (notational) representations of numbers. In many contemporary countries, numerals for numbers larger than nine use the place-value principle. In any place-value numeral system, the same symbol represents different quantities depending on its location in a string. The commonly used Western numeral system [1] has 10 elements (‘0’, ‘1’, ‘2’, ‘3’, ‘4’, ‘5’, ‘6’, ‘7’, ‘8’ and ‘9’) and a base-10 place-value system. So the numerical value of the last digit read in a string is the digit × 1 (10^0^), the value of the second digit from the end is the digit × 10 (10^1^), the value of the third digit from the end is the digit × 100 (10^2^), etc.

*Numerical expressions*. In this review, we focus on spoken and written (verbal) numerical expressions. All verbal numeration systems contain a limited lexicon for units (e.g. ‘one’ to ‘nine’ in English). In languages with a decimal system, this lexicon also includes a word for the base (e.g. ‘ten’ in English) and its powers such as ‘hundred’, ‘thousand’. Such words are called ‘lexical primitives’ or ‘atoms’ according to the Glossary of Pelland [2]. Typically, these elements can be combined following a syntax. In English and related languages, this syntax can have additive (e.g. in English 349 is spoken as ‘three hundred (and) forty-nine’: 300 + 49) and count-based rules (e.g. in English 500 is spoken as ‘five hundred’: 5 (×) 100). Children typically master count-based before additive rules [3]. While Western numerals are widely used across different cultures and languages, numerical expressions are language-specific and other languages may have additional and/or alternative rules [4]. In line with the available research, the main focus of this review will be on languages with a decimal system whose lexicon includes atomic expressions for units and still relatively compact ones for decades. For examples from a wider range of verbal systems with diverging structures, see for instance Barlow [4] and Verkerk *et al.* [5].

*Prior knowledge*. Children typically learn numerical expressions before Western numerals. Critically, they eventually encounter numerals mostly in the context of already familiar numerical expressions. In many educational situations, prior knowledge is activated where it is irrelevant and perhaps even unhelpful. One example is the *natural number bias*: learners’ knowledge about natural numbers influences how they process rational numbers: for instance, many children think that 1/56 is larger than 1/6, because 56 > 6. Interestingly, prior-knowledge structures can coexist with the new-knowledge structures, and interfere with them, for many years [6–8]. Because, in many cultural contexts, numerical expressions and numerals have different properties (often related to bases, as discussed below), and because they are encountered at different points across development, there is the potential for prior knowledge about numerical expressions to influence how Western numerals are processed. Exactly how these effects manifest varies across languages.

## 2. Cognitive models of number processing

Cognitive models of human number processing typically acknowledge the importance of different formats of number representations—specifically, numerical expressions and Western numerals for exact numbers—and agree that numerical behaviour is influenced by the relationship between these representational formats. But they do so in different ways. Here, we briefly introduce the three most relevant theoretical accounts of number processing: the *triple code model*, the *single semantic route model* and *ADAPT*. In the discussion, we will reflect on how these models could be adapted to account for the findings reviewed below.

In the *triple code model* [9,10], three formats are postulated: an analogical representation, a verbal format and a visual number form. In the analogical representation of quantities, numbers are represented as distributions of activations on a mental number line [9]. In the verbal format, numbers are represented as numerical expressions (e.g. ‘fifteen’), and in the visual number form as strings of digits (e.g. ‘15’). These three codes are directly interconnected. Although there is no abstract semantic component assumed, the analogue magnitude component has a semantic function. Transcoding between numerical expressions and numerals is achieved by using either the direct asemantic route between the verbal format and the visual number form codes, by using the indirect semantic route involving the analogical magnitude representation, or by using both routes concurrently. The model makes no explicit predictions about the role of the base or the structure of numerical expressions.

In contrast, in the *single semantic route model* [11] number processing relies on a single amodal representation and three functionally distinct modules. Dedicated mechanisms exist for each type of format (spoken or written numerical expressions or Western numerals). All number formats are converted into modality-neutral abstract representations which specify the magnitude of the number. The abstract representation expresses the numerical quantity in a proposition-like language by using powers of 10. The Western numeral ‘1989’, for example, and the verbal numeral ‘one-thousand-nine-hundred-eighty-nine’ are both formally expressed as {1} 10^3^, {9} 10^2^, {8} 10^1^, {9} 10^0^. This model thus proposes that the abstract internal number representation is in base-10 format, and one of its main assumptions is that these abstract representations underlie all numerical processes, including number transcoding and calculation.

*ADAPT* [12] (a developmental, asemantic and procedural model for transcoding), finally, aims to account for the way children learn to transcode from a spoken numerical expression to a numeral. It proposes that children and adults can transcode without accessing numerical meaning. First, the spoken numerical expression is parsed sequentially and segmented into units which are stored in working memory (WM). Relevant matching knowledge (entries of lexical primitives such as the unit, ‘-teen’, etc. and their digital forms) from long-term memory (LTM) is then activated in WM, with learnt transcoding procedures that are applied to construct WM representations of a sequence of digits. ADAPT conceptualizes development as a combination of an increase of entries in LTM, the adding of new procedures, and an increase in WM. While the numerical expressions and digit string entries in LTM for the original ADAPT version are in base-10, ADAPT could apply to any numerical base.

## 3. The acquisition of numerical expressions is influenced by base transparency

In this section, we first focus on the typical chronological acquisition of spoken number words and numerical expressions in contemporary children growing up mainly in an Indo-European language context. Base transparency only comes into play for numerical expressions that are larger than or equal to the base. However, the first numerical expressions children learn are typically below the base, and those are in turn constituents of the larger numerical expressions above the base. Thus, we start with the acquisition of numerical expressions below the base before introducing base transparency. When comparing the acquisition of spoken number words and numerical expressions of children growing up in different language contexts, however, it is usually not only the degree of base transparency that differs between languages (and children’s learning context). We end this section by reflecting on potential confounds in the existing research.

### (a) Findings

Findings differ for different ranges (up to the base, between 10 and 20, and beyond).

*Up to the base*. Spoken numerical expressions are the first exact numerical concepts children encounter. In general, children learn numerical expressions earlier the more frequently those numerical expressions are used in common everyday speech. The numerical expressions ‘one’ to ‘ten’ have high frequencies in languages with a decimal system [13]. In such languages, numerical expressions for the smallest whole numbers, e.g. 1 to 10, tend to be unique words that have to be learnt item by item. Following Pelland [2], we call these number words and other lexical primitives ‘atoms’.

Children can produce spoken numerical expressions without understanding their meaning. Young children can often recite a count list verbally long before they know the exact numerical value of each count word. Similarly, if children are given four objects, they might be able to point to each object in turn to count ‘one’, ‘two’, ‘three’, ‘four’, but might still not understand that the last numerical expression of the count sequence describes the total quantity of the set. Understanding this so-called cardinality principle is an important milestone in children’s numerical development [14].

The actual numerical expressions that a child learns depend on the language background of the child. However, the acquisition of numerical expressions in languages with decimal systems should be similar for numbers up to 10 as such languages tend to have exactly 10 atomic ordered words that need to be learnt. Cross-cultural studies investigating counting skills in children aged between 3 and 6 years suggest that, for numbers up to 10, average performance is indeed similar in English, French and Chinese [15–17].

**Figure 1.**
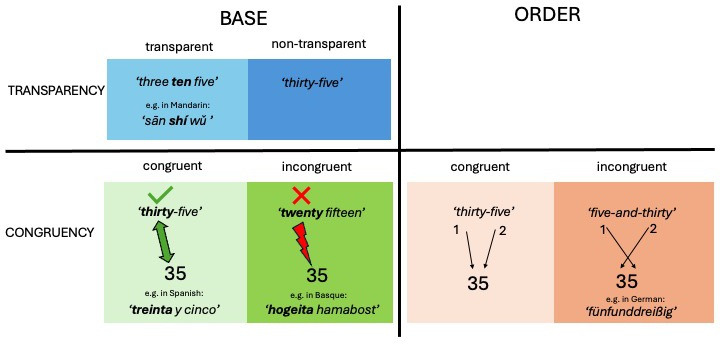
Examples of numerical expressions that are transparent versus non-transparent with respect to base and congruent versus incongruent with respect to base or order.

*Beyond the base*. Numerical expressions above the base are often constructed from building blocks (i.e. the base and other atomic number words [2]), which may be shared across different numerical expressions. In decimal languages with highly regular construction of numerical expressions such as Japanese and Mandarin, once children have learnt the number words for 1 to 10, they can combine these elements to create all numerical expressions for numbers up to 99. For example, in Mandarin 11 is ‘shí yī’ ( = 10_1), 21 is ‘èr shí yī’ ( = 2_10_1) and 91 is ‘jiǔ shí yī’ ( = 9_10_1). In such languages, the base-10 system is clearly and systematically marked in the numerical expressions for numbers from 11 onwards, which is why we call these expressions *base transparent* (figure 1).

In contrast, many languages use more opaque rules when generating numerical expressions beyond 10. For instance, the morphemes used to construct decade words larger than 10 in English (‘-ty’) or German (‘-zig’) are only opaquely related to the word for 10 (‘ten’ and ‘zehn’, respectively) and are therefore less base-transparent. Furthermore, often the decade multiplier, at least for the first few decade words, is a variation of the unit word, e.g. ‘twenty’ instead of ‘twoty’ and ‘thirty’ instead of ‘threety’ (but ‘forty’, ‘fifty’ …) in English or ‘zwanzig’ instead of ‘zweizig’ and ‘dreißig’ instead of ‘dreizig’ (but ‘vierzig’, ‘fünfzig’) in German. French has decade words that deviate from base-transparency in an additional way for 70 (‘soixante-dix’ = 60_10), 80 (‘quatre vingt’ = 4_20) and 90 (‘quatre vingt dix’ = 4_20_10). These inconsistencies in how the numerical building blocks are used for numbers beyond 10 influence the acquisition of numerical expressions and subsequent understanding, as was shown in a study with English- and Korean-speaking children. The children were shown material organized in four sets of 10 items and asked to give the experimenter a particular number of those items (e.g. 32 items). Children from a base-transparent language background (Korean) retrieved sets of 10 items each plus individual items (i.e. 10 + 10 + 10 + 2 items) at a younger age than children from a less base-transparent language background (English) who were mostly still counting out individual items [18].

*Between 10 and 20 (the ‘-teens’)*. In many languages with decimal systems (including Indo-European languages [19] such as English, French, Hindi, Russian, Swedish, but also others such as Arabic), the numerical expressions for numbers between 10 and 20 (‘-teens’) show even more irregularities than numerical expressions for 20 and above. Many numerical expressions for ‘-teens’ are *not base transparent*. First, the lower numbers in this range are often expressed with atomic number words, not explicitly indicating the range (e.g. ‘eleven’ and ‘twelve’ in English). Second, usage of inverted numerical expressions is more frequent in this range than beyond 20 (e.g. English: ‘fourteen’ instead of ‘ten four’; Polish: ‘jedenaście’ = 1_10 [20]). Third, the construction of numerical expressions for ‘-teens’ within a language can be inconsistent (e.g. Italian: ‘undici’ = 1_10 but ‘diciotto’ = 10_8). This is a source of considerable difficulty for children. Rather than simply applying a consistent rule for numbers larger than 10, they must deal with irregularities that do not facilitate the acquisition of numerical concepts.

Several studies investigated the acquisition of such numbers in different languages and reported a delay in the acquisition of numerical expressions for numbers larger than 10 for languages for which numerical expressions are less transparent [15–17,21,22]. For instance, Miller *et al.* [16] compared early counting skills in 3–5 years old English- and Mandarin-speaking pre-schoolers. While there were no differences in counting skills between language groups for numbers up to 10, significant language differences favouring Mandarin-speaking children emerged in the range between 10 and 20, with language differences in counting being most pronounced for numbers above 12 [16]. These and other findings [23,24] provide evidence that children from a language background with numerical expressions for ‘-teens’ that are not base-transparent need more time to master number names for numbers beyond 10.

### (b) Potential confounds

It might be tempting to take this as evidence that base-transparent numerical expressions are easier to learn and process. However, the evidence from natural languages does not allow us to disentangle whether the explicit presence of the base in the numerical expressions or the systematic use of generative transparent rules facilitate the learning of numerical expressions.

*Influence of languages versus cultural and educational differences*. Cross-linguistic comparisons are often cross-cultural as well as cross-country comparisons, where children are from different educational systems. Thus, differences in, for instance, exposure to numerical expressions and counting [24], approaches to teaching and learning [21], as well as differences in home experiences [15] need to be considered as additional or alternative explanations for differences in the acquisition of numerical expressions and counting.

One way to ameliorate such issues with cross-cultural studies is to choose samples so that factors other than language are constant. Mark & Dowker [25], for example, investigated linguistic influences on mathematical development in the same culture and educational system (for the same within-culture approach, see also [26–30]). They compared children who spoke Chinese at home and learnt to count in Chinese at school with children who spoke Chinese at home and learnt to count in English at school. Results showed that the language in which numbers are taught does play a role, however, only for some tasks (counting backwards, numerical skill test) but not for others (counting forward, place-value understanding).

## 4. Mapping between numerical expressions and numerals is influenced by the transparency and the congruency of base and the congruency of order

In this section, we first describe some of the hurdles children have to overcome when they are learning how to write multi-digit Western numerals. Then, we move on to discuss how the relationship between numerical expressions and Western numerals might influence numerical processing and development, and focus on three aspects that affect the ease of that mapping.

Multi-digit Western numerals are typically taught explicitly to children during their first few years of schooling. In many languages, the mapping from spoken numerical expressions to multi-digit Western numerals is not straightforward. Chrisomalis [31] states that there is always some but never complete correspondence between the numerals and the spoken numerical expressions in the language of the society where the system was invented. For example, zeros are absent in spoken numerical expressions, but essential in the Western numerals (compare for instance English ‘four hundred three’ and ‘403’) [3]. Overproduction of zeros in number writing (e.g. ‘4003’) is a common mistake in children in pre-school [32,33] and in the first years of schooling [34,35]. This clearly shows the influence of prior knowledge—specifically about the structure of spoken numerical expressions—on children’s ideas of written numerals.

When children acquire the Western numeral system, they map their existing knowledge of numerical expressions onto the new multi-digit numerals. Three important aspects influence the ease of mapping: *transparency of base, congruency of base* and *congruency of order* (figure 1).

For example, the mapping between numerical expressions and multi-digit numerals is both base transparent and base and order congruent in many East Asian languages [36,37]. The mapping is *base transparent* because the base (10) is consistent and present in numerical expressions, providing relevant information about the place of a number symbol in the multi-digit numeral (e.g. for 42 = ‘
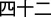
’_= ‘sì shí èr’ = 4_10_2, indicating the 4 to be a multiplier for 10). The mapping is *order-congruent* because the order of the elements in the numerical expression corresponds to the order of the digits when read from left to right. The concurrent presence of base transparency and order congruency is rare in many Western languages (with few exceptions, e.g. modern Welsh [27]). In the following paragraphs, we will give an overview of studies that have investigated the impact that a lack of transparency and congruency between numerical expressions and numerals might have. In these studies, both children and adults have been tested across a broad range of numerical and mathematical tasks involving multi-digit numbers. While samples and methods in these studies are varied, it is clear that the impact of a lack of transparency and/or congruency is not a transient phenomenon.

*Transparency of base*. The impact of base-transparent mapping is illustrated by cross-cultural studies that investigated possible advantages of highly base-transparent systems of numerical expressions (e.g. in Japanese, Korean or Chinese). Base transparency is linked to better place-value understanding in pre-schoolers and first graders [37–39]. For instance, Japanese and Korean first graders preferred constructing multi-digit numbers using 10- and one-blocks, whereas American, Swedish and French children used collections of one-blocks, indicating a less advanced understanding of the base-10 place-value structure in children with the less base-transparent number system [37]. The impact of base transparency was also investigated in other, more complex numerical tasks. While some such studies found a beneficial effect for children (e.g. in addition [40,41]), others did not (e.g. not in number line estimation [42–44] and/or not in addition [23]). However, base transparency is not the only base-related issue which might influence such tasks. For instance, Schlimm & Neth [45] systematically compared the efficiency of Western and Roman numerals for basic arithmetic operations. Because the Roman system has fewer symbols, the LTM requirement for arithmetic facts is lower in that system, but the number of operations needed to complete various arithmetic algorithms is typically higher. Similar analyses were offered by Bender & Beller ([46], see also [47]) for the mixed-base Mangarevan numeration system. However, to our knowledge, empirical studies of how the properties of such systems influence human behaviour have yet to be done.

Is it the presence versus absence of base transparency—rather than other factors—that causes the observed differences between cultures? The impact of differences in teaching and learning approaches, for example, may grow with increased exposure to formal schooling. That differences in place-value understanding [37] and addition [40] are evident *before* these concepts were explicitly taught in school seems to suggest that base transparency indeed plays a role during the early acquisition of these concepts.

*Congruency of base: base-10 versus base-20*. Insights into the effect of congruency of base can be gained from languages with a base-20 system for numerical expressions. In French, numerical expressions for numbers from 80 to 99 are constructed by drawing on base 20 (e.g. 82 = ‘quatre-vingt-deux’ = 4_20_2). Van Rinsveld & Schiltz [48] investigated the effects of this base-20 structure in French numerical expressions. They found that English-speaking children showed better transcoding performance than French-speaking children, but only for numbers larger than 70. Such processing costs for numerical expressions with a base-20 structure have been replicated in a within-subject design with French-German bilingual children and adults for both transcoding [49] and addition problem solving [50].

To investigate the impact of a *genuine* base-20 system on addition, Colomé *et al.* [26] worked with Basque- and Spanish-speaking adults. In Basque the numerical expression for, say, 35 is ‘hogeita hamabost’ (= 20_15). Colomé *et al.* therefore manipulated visually presented addition problems so that problems either matched (e.g. ‘20+15 = ’) or did not match (e.g. ‘25+10 = ’) the base-20 structure. Basque speakers were specifically faster than Spanish speakers when addends followed the same vigesimal structure as the Basque numerical expressions. In other words, while (irrelevant) prior knowledge about a number word system that does not (consistently) use base-10 often interferes with performance on tasks involving base-10 numerals, it may also speed up processing in those contexts in which the numerical expressions map onto the structure of the problem at hand.

*Congruency of order*. The lack of congruency of order in numerical expressions—also typically referred to as number word inversion—hinders mathematical development and also affects adult numerical processing. For example, children speaking languages with inverted numerical expressions up to 99 (e.g. German, Dutch) commit more overall transcoding errors than children speaking languages with mostly non-inverted numerical expressions (e.g. French, Italian) ([30,51], but see [29]). Furthermore, showing the direct effect of the inverted numerical expressions, up to 50% of their transcoding errors are inversion-related errors (e.g. writing ‘42’ instead of ‘24’ for ‘vierundzwanzig’ (4_20) [29,30,51]. Number word inversion has also been shown to impact performance in children and adults in magnitude comparison tasks with Western numerals [52,53], addition verification with Western numerals [54] and addition production when problems are presented as numerical expressions and the results typed in as Western numerals [55]. Across tasks, performance differences are typically attributed to the increased demand when trying to map numerical expressions that are not order-congruent. Crucially, the specificity of the predictions of the influence of order-incongruency on performance often helps to exclude that the effects are purely driven by general cultural differences.

That transparency of base and congruency of base and order influence the accuracy of number transcoding, i.e. number reading and writing [34], and number matching in children [56] and even healthy adults [57] is perhaps not surprising. However, transparency of base and congruency of base and order are also crucial in the *absence* of overt numerical expressions, e.g. for magnitude judgement of multi-digit numerals [58,59], and they affect the ease of acquisition of further, more advanced mathematical skills and concepts (e.g. understanding the place-value principle of multi-digit numerals, solving multi-digit arithmetic problems [54]). This highlights that even highly automatized processing of multi-digit numerals in adults shows traces of the structure of numerical expressions in people’s native language [60] and provides evidence that number processing is not entirely language-independent, even in skilled adults.

## 5. Implications for learning and instruction

Overall, the lack of transparency of base, of congruency of base and of congruency of order in the structures of numerical expressions presents a hurdle for numerical processing and learning [61]. Suggestions on how to help learners to compensate for the increased complexity of non-transparent or incongruent systems of numerical expressions are rather scarce and often not tested with rigour. A radical suggestion towards more base transparency could be to reform the curriculum by skipping the range between 10 and 20, and instead to first introduce children to decade names and numerical expressions beyond 20. This may allow them to grasp the systematicity of the base-10 place-value system before introducing the typically highly irregular numerical ‘-teen’ expressions [62].

Norway addressed the issue of numerical expressions beyond 20 that are not order congruent in 1951 when the country changed an inverted number word system beyond 20 to a more regular system of numerical expressions nationwide [63]. The reason for this change was quite practical in nature: telephone numbers changed from five- to six-digit numbers, and the Telegraph Agency had noticed that fewer errors were made when the numbers were pronounced in the same order as they were written. To date, the old system of numerical expressions is still in use (especially among older generations) alongside the new order-congruent system. However, it is hard to quantify the impact of this change across generations.

An alternative might be to use adapted, more regular numerical expressions in early learning (e.g. for the ‘-teens’ in English: ‘-ty’ with ‘one-ty’, ‘one-ty one’, ‘one-ty two’, … for 10, 11, 12, respectively; and decades: ‘two-ty’, ‘three-ty’ and ‘five-ty’ for 20, 30 and 50, respectively) [61]. A few empirical studies have investigated whether replacing numerical expressions that are not base transparent with base-transparent ones during mathematics lessons may have a positive impact on children’s numerical and mathematical learning [62,64–66]. For example, English- and Spanish-speaking children who learnt to count in pre-kindergarten with base-transparent numerical expressions (‘one-ten one’, ‘one-ten two’ … for 11, 12 …) showed significantly higher mathematical achievement [65]. However, while results of these studies are promising, further, well-powered experimental and longitudinal studies are needed.

## 6. Non-decimal bases in numeral systems

As discussed above, many cultures use non-decimal numerical expressions alongside Western base-10 numerals, leading to a lack of congruency of base. For example, Basque speakers refer to 35 using both the numeral ‘35’ and the word ‘hogeita hamabost’ (20 15). But there are also a great many non-decimal numeral systems (for a comprehensive overview, see Chrisomalis [31]). How are these understood? Surprisingly, there is relatively little research on the topic.

*Binary (base 2) and hexadecimal (base 16) systems*. Understanding numerals represented in a place-value system with non-decimal bases is of interest for teaching computer science. Goldman *et al.* [67] conducted a Delphi study to identify concepts that computer science students should learn in undergraduate logic design courses. Participants agreed that number representation—which the authors defined to be ‘understanding the relationship between representation (pattern) and meaning (value)’—is one of the 10 most important concepts. Given this, it is unsurprising that several researchers have designed and evaluated novel teaching techniques that aim to help students come to understand and operate with numerals represented in a place-value system with a base other than 10. For example, Kordaki [68] conducted a small-scale evaluation of a computer-based card game—essentially a binary version of blackjack—designed to help elementary school children understand base 2, and found ‘encouraging’ results. Kempthorne & Steele [69] compared three different teaching techniques designed to help computing undergraduates convert between decimal, binary and hexadecimal numerals. They found that teaching in groups, in traditional lectures and using game-based learning all yielded comparable outcomes on a subsequent test. Like in Kordaki’s [68] study, their sample size was relatively small, and neither study used an experimental design with a control group.

Few computer science educators have explored the nature of the difficulties students have when engaging with non-decimal bases. An exception is Herman *et al.* [70], who conducted interviews with 26 undergraduate students and administered a paper and pencil test to a further 700. They identified a series of misconceptions that students exhibit when working with non-decimal place-value numerals. For example, some students do not understand that changing the base changes the weights associated with each position in the numeral (e.g. that the 2 in the hexadecimal numeral 2A represents 2 × 16 not 2 × 10). Around 15% of Herman *et al*.’s [70] large sample reliably exhibited behaviour consistent with this misunderstanding. Similarly, numbers represented in larger bases are not necessarily larger than those represented in smaller bases, although around 25% of students responded to items on Herman *et al*.’s test as if this were the case. For instance, one student (correctly) wrote ‘(11)_2_’< ‘(14)_10_’ < ‘(15)_16_’ (using notation where the subscript denotes the base, so this expression is equivalent to the base 10 expression ‘3’ < ‘14’ < ‘21’) while (incorrectly) stating that ‘obviously binary is small, so it’s smallest, and then decimal is smaller than hex’ (p. 296). Another noted, when comparing (1.1)_2_ and (1.5)_16_, ((1.5)_10_ and (1.3125)_10_), that ‘a one in base 16 is obviously bigger than a one in base 2, no matter what’s after [the point]’ (p. 297). In other words, some students apparently believe that numbers represented in larger bases must be larger. During Herman *et al*.’s interviews some students used improper positional weights, especially in the units’ place. One student, for example, interpreted the B in the hexadecimal number (2B)_16_ = (43)_10_ as representing 11 × 16^1^ rather than 11 × 16^0^. Positional issues particularly manifested during subtraction questions, when students found it hard to know how much to ‘borrow’ from subsequent columns. In sum, many computer science undergraduates find dealing with non-decimal place-value number representations difficult, and these difficulties follow systematic patterns.

*Bases in artificial numeral learning*. Studies on this issue from the numerical cognition literature show a different picture. Both Krajcsi & Szabó [71] and Weiers *et al.* [72] used an artificial numeral learning paradigm to compare the learning of place-value systems (e.g. Western numerals) and sign-value systems (such as Roman numerals), both with the same non-decimal base, and found that adults were surprisingly good at learning novel place-value systems.

**Figure 2.**
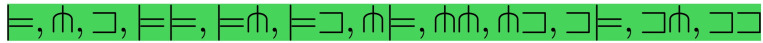
The first 12 numerals in an artificial base-3 place-value number system (without a zero).

Weiers *et al.* [72] assigned 204 adults to receive either training in an artificial base-3 place-value system or an artificial base-3 sign-value system. Participants were trained in the new system using an ordinal approach: the first 12 numerals of the system were shown in turn a total of 60 times. For example, participants in the place-value condition might have repeatedly been shown the sequence shown in figure 2.

**Figure 3.**
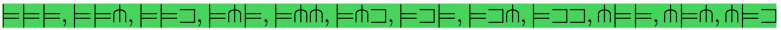
The next 12 numerals following on from those in figure 2.

Was this training sufficient for participants to understand the relative numerical values of numerals from the new system? To test this, Weiers *et al.* asked participants to perform 528 symbolic comparisons where participants were presented with two numerals and asked to choose the larger. Crucially, during the comparison task, participants were presented not only with the 12 learnt numerals, but also the next 12 in the system, as shown in figure 3.

Participants succeeded on this task with relative ease (mean accuracy 81% for learnt numerals and 76% for non-learnt numerals).

Krajcsi & Szabó [71] used an explicit learning paradigm, where the rules of the system were explicitly taught. They found even higher accuracy rates (>95%) on their comparison task.

Whereas Weiers *et al*.’s [72] and Krajcsi & Szabó’s [71] participants were apparently able to learn basic features of novel non-decimal number systems with relative ease, the computer science students studied by Herman *et al.* [70] had systematic difficulties. What accounts for this difference? There are at least two possibilities.

First, both Weiers *et al.* [72] and Krajcsi & Szabó [71] adopted the standard approach from the numerical cognition literature of measuring their participants’ understanding of the numerals using a magnitude comparison task. While magnitude is clearly a central property of numbers, it is not the only one. In contrast, Herman *et al.* [70] used more sophisticated tasks involving the translation between bases and operations such as addition and subtraction. Perhaps Weiers *et al*.’s and Krajcsi & Szabó’s participants would also have struggled if faced with these more complex tasks.

Second, while Herman *et al.* used traditional Western numerals that their participants were familiar with, both Weiers *et al.* and Krajcsi & Szabó used novel artificial symbols. As noted above, prior knowledge is often activated in inappropriate situations (e.g. creating a natural number bias). In other words, whenever a familiar symbol has to be dealt with in a new way, prior knowledge will need to be inhibited, a cognitively demanding task. Dealing with non-decimal number systems using familiar symbols could be so difficult for learners because it requires the inhibition of knowledge of the decimal system.

## 7. Questions for future research

In sum, our review shows that the two most common ways to represent exact numbers (numerical expressions and Western numerals) give rise to problems when mapping one onto the other is not congruent. Especially in educational settings, prior knowledge can then be activated in inappropriate situations in a manner that interferes with mathematical learning. Three important areas for further cognitive research include the revision of theories to cover the usage of multiple systems, designing methodological strategies for disentangling confounding factors, and a more in-depth investigation of how prior knowledge gets activated—and can be inhibited.

*Integrating considerations of base into theories of numerical cognition*. It is notable that the three theoretical accounts we discussed at the beginning of this article—the triple code model, the single semantic route model and ADAPT—did not reemerge during our discussion of existing cognitive work on numerical bases. In general, work on numerical cognition tends to assume that humans are monolingual and monosystem. However, multilingualism is common and over half of the world’s population use several languages in everyday life [73]. As other articles in this issue demonstrate [31,47,74], the assumption of monolingualism and monosystem use misses not only important numerical phenomena, but also opportunities to test and refine existing models. For instance, the triple code model posits verbal and visual number formats, but would a bilingual, bisystem individual be more appropriately conceptualized as having five (or more) codes, or three enriched codes? Would the individual’s various systems activate analogical representations to a similar degree, and would refinement to the acuity of these analogical representations equally impact the different verbal and visual codes? Would the strength of the asemantic routes between same-base verbal/visual representations be comparable to the strength of the asemantic routes between different-base verbal/visual representations? We know very little about these questions because the blind spots of the models prevented the empirical investigation of such questions. However, a bilingual triple code model has been proposed recently [75] and future research will have to test its validity in a variety of contexts.

*Exploring the contribution of specific inconsistencies*. For any two natural languages there is often more than just one difference between the structure of their numerical expressions (e.g. two languages might differ in their number of atomic numerals and the transparency of their decade multipliers). Teaching participants new artificial systems of numerical expressions and/or notational numerals might be an elegant solution to isolate the impact of specific inconsistencies for at least three reasons. First, several artificial systems can be created, manipulating the specificities between these systems separately, element by element (such as number of atomic numerals, consistency of teen/decade markers, transparency of decade multipliers, inversion, etc.). Then the effects of particular elements on learning can be compared in isolation.

Second, another advantage of teaching artificial systems is that they can be constructed in such a way that they are entirely novel symbols and independent of the Western numeral system and its constraints: for instance, all symbols can be novel symbols, thus meaningless prior to learning and/or the size of their base can vary. This will allow for investigating whether for example base-10 is easier to learn than base-3 when just learning a new symbol system, i.e. a set of written symbols, in isolation, without learning corresponding numerical expressions.

Third, and most importantly artificial numerical expressions and corresponding artificial numeral symbols can be taught in the same study, mimicking the natural number learning process while separately manipulating isolated elements of interest.

Regardless of the variant(s) of artificial systems implemented/taught participants would still be affected by prior knowledge linked to the structure of the numerical expressions in their native language (and likely also by the greater familiarity with a base-10 system). It would thus be essential to also run these artificial number experiments cross-linguistically with speakers of carefully chosen languages.

*Effect of prior knowledge*. As noted above, many of these issues can be conceptualized as resulting from conflicts between knowledge activated in the context of numerical expressions and knowledge generated in the context of numerals (see also Bender [47], for further examples of conflicts that arise from parallel usage of systems in different representational formats and with diverging properties, including bases). Research in other situations demonstrates that such conflicts can be surprisingly long-lasting. For example, even research-active mathematicians show traces of natural number bias in their reaction times [7], but individuals also vary systematically in their ability to inhibit such irrelevant information [76].

Are there individual differences in children’s and adults’ ability to inhibit prior knowledge in the context of numerals and numerical expressions? For example, some speakers of German or Dutch might show consistently smaller number word inversion effects than other speakers of the same languages. Such a possibility seems plausible: it is well established that measures of children’s inhibitory control skills often correlate with their mathematics achievement [77,78]. Understanding such individual differences and what strategies or cognitive resources high-inhibition individuals recruit when faced with conflict situations might assist in designing interventions that could benefit children.

## 8. Conclusion

Most empirical research that can inform our understanding of the effect of numerical bases on numerical processing has been carried out with speakers of Indo-European and East Asian languages using mostly base-10 systems and Western numerals. However, the type of system used is a critical factor for cognitive processing. For instance, the acquisition of numerical expressions is easier, and numerical understanding is typically more advanced, in children acquiring languages that use base-transparent numerical expressions. Similarly, transparent mapping of base and congruency of base and of order between numerical expressions and Western numerals leads to more efficient numerical processing. Whereas several factors are confounded in such comparisons, artificial number systems might be a promising method for systematic future research because they allow for singling out and directly manipulating features of interest. However, even in artificial learning paradigms, learners’ prior knowledge (e.g. with a particular language or a base-10 system) will influence their learning. Future research and theoretical developments therefore need to go beyond ‘standard’ study populations and move away from conceptualizing learners as monolingual and monosystem because a major part of the world’s population lives in a multilingual context.

## Data Availability

This article has no additional data.
